# Skin manifestations and related clinical characteristics of multisystem inflammatory syndrome in children: A descriptive retrospective cohort study at Texas Children’s Hospital

**DOI:** 10.1016/j.jdin.2024.09.009

**Published:** 2024-10-22

**Authors:** Matthew Penna, Lauren Pupa, Grace Lee, Soo Jung Kim

**Affiliations:** a Department of Dermatology, Baylor College of Medicine, Houston, Texas; b Department of Dermatology, Texas Children’s Hospital, Houston, Texas

**Keywords:** clinical research, COVID-19, multisystem inflammatory syndrome in children, pediatric dermatology, rash, SARS-CoV-2

## Abstract

**Background:**

Little is known about the dermatologic manifestations of multisystem inflammatory syndrome in children (MIS-C) in children and adolescents.

**Objective:**

We sought to describe the demographic background, key clinical features, and the clinical consequences of developing rash manifestations in MIS-C patients at Texas Children’s Hospital.

**Methods:**

Descriptive retrospective cohort study of 290 hospitalized eligible patients between May 2020 and April 2022.

**Results:**

Among MIS-C patients, 51% exhibited a rash. We found that younger children (8.62 vs 9.49 years of age, *P* = .006) and White children (*P* = .002) had a higher likelihood of developing a rash in association with MIS-C. Additionally, patients without a rash had increased maximum troponin levels (0.11 ng/mL vs 0.07 ng/mL, *P* = .02) and a higher incidence of cardiac involvement (83.1% vs 72.3%, *P* = .03) compared to those with a rash but did not significantly affect the length of hospital stay or clinical course. The most commonly observed rash was an erythematous and maculopapular rash on the trunk and/or extremities.

**Limitations:**

Rash characteristics were initially described by a variety of physicians in the pediatric primary care services.

**Conclusion:**

Rash manifestations in MIS-C patients are associated with lower cardiac involvement and decreased troponin levels.

**Capsule Summary**

• Little is known about the clinical consequences of developing a rash in relation to multisystem inflammatory syndrome in children (MIS-C).
• This study describes the dermatological manifestations associated with MIS-C and provides useful information for clinicians on the demographics and clinical sequelae of MIS-C patients who develop a rash.

## Introduction

Multisystem inflammatory syndrome in children (MIS-C) is a severe illness that has emerged in association with SARS-CoV-2 infection. The Centers for Disease Control and Prevention define MIS-C as a condition affecting individuals below the age of 21, characterized by fever, laboratory indicators of inflammation, and severe clinical symptoms that necessitate hospitalization.^1^ The condition involves the dysfunction of 2 or more organ systems and cannot be attributed to any other known infectious or noninfectious cause. Since the emergence of SARS-CoV-2, there has been an increased incidence of MIS-C cases worldwide with COVID-19 implicated in the rising incidence.^2^ During the initial stages of the COVID-19 pandemic, the incidence of MIS-C was approximately 1 in 3000-4000 children who contracted SARS-CoV-2.^3^ The occurrence of MIS-C has declined over time with the emergence of vaccinations and increased immunity to SARS-CoV-2; however, much of the pathophysiology for MIS-C remains largely unknown.^4^

MIS-C is more commonly observed in boys and disproportionately affects Black/African American children.^5^ The affected organ systems vary but typically include a combination of cardiac, renal, respiratory, hematologic, gastrointestinal, dermatologic, or neurological systems in varying degrees. One study reported that 73% of MIS-C patients had mucocutaneous involvement, with the most common finding being erythema or rash (59%).^6^ The rashes can present in various forms, including maculopapular, urticarial, or vesicular. Other mucosal findings reported included oral mucosal changes (65.1%), conjunctival injection (58.1%), and erythematous, fissured lips (27.9%).^7^

This study aims to explore the relationship between rash manifestations and the emergence of MIS-C among a diverse patient population seeking medical care at Texas Children's Hospital (TCH), one of the largest and most prominent pediatric hospitals in Texas. We were particularly interested in the demographic and clinical differences in the MIS-C patients who developed cutaneous manifestations in the form of a rash versus those who did not. By thoroughly detailing MIS-C-related outcomes, this study seeks to add to our understanding of the unique presentation of MIS-C, particularly in association with the development of a rash as part of the disease course.

## Methods

A descriptive retrospective cohort study was conducted on patients younger than 21 hospitalized with suspected MIS-C between May 2020 and April 2022 at TCH in Houston, Texas. To be included in the final analysis, patients had to meet the Centers for Disease Control and Prevention definition of MIS-C; MIS-C patients must be younger than 21 years old with fever and laboratory evidence of inflammation involving at least 2 organ systems, along with suspected or confirmed exposure to COVID-19 within the previous 4 weeks or positive test results indicating SARS-CoV-2 infection (reverse transcription-polymerase chain reaction, serology, or antibody testing). Patients with alternative diagnoses were excluded. In this study, we analyzed patient data from 317 suspected cases of MIS-C at TCH between May 2020 and April 2022. This study includes 290 patients after excluding 27 patients who did not meet the definition criteria or had previously been diagnosed with MIS-C. This study was approved by the Baylor College of Medicine Institutional Review Board (H51919).

Data collection was obtained from electronic medical records and was based on physical exam findings, laboratory values, and treatment plans by primary pediatric inpatient physicians. Severe rashes were seen and classified by pediatric dermatologists at TCH to rule out Steven Johnson syndrome and drug rash with eosinophilia and systemic symptoms. Given the severe morbidity associated with MIS-C, the majority of patients were managed by the primary care team, rheumatologist, and organ-system-specific consultation. For this study, pediatric dermatologists were involved in the retrospective classification of rashes in patients based on photos that were not directly managed by the dermatology team while hospitalized.

Continuous data of demographic and clinical characteristics were analyzed using the Wilcoxon rank-sum test, while categorical data were analyzed with Fisher's exact test. Statistical significance was determined with a threshold of *P* < .05. Statistical analyses were performed using Prism software (version 8.0, GraphPad).

## Results

We found that 24.1% (70/290) of patients had positive reverse transcription-polymerase chain reaction tests upon admission, while 96.2% (279/290) had reactive SARS-CoV-2 (IgG + IgM) antibodies. Nearly all patients had previously contracted COVID or had been in contact with a COVID-19 positive patient within the previous month. Among the 290 patients diagnosed with MIS-C, 24.1% (70/290) were diagnosed from May 2020 through December 2020, 35.5% (103/290) were diagnosed from January 2021 through July 2021, and 40.3% (117/290) were diagnosed from August 2021 through April 2022.

The median age at admission was 9.26 years (interquartile range [IQR], 5.91, 12.58 years) (Table I). The majority of MIS-C patients were male (57.2%, 166/290). Additionally, we found that 62% (180/290) of MIS-C children were White, 27.6% (80/290) were Black/African American, 6.2% (18/290) were Asian, 45.9% (133/290) identified as Hispanic or Latino, and 6.9% (20/290) did not report their race/ethnicity or reported “other.” Moreover, 85.2% (247/290) of all MIS-C patients were previously healthy with no known comorbidities.

**Table I:** Demographics of MIS-C patients with and without a rash

Demographics and disease course	MIS-C without rash involvement (*n* = 142), no. (%)	MIS-C with rash involvement (*n* = 148), no. (%)	*P* value	Odds ratio (95% CI)	All MIS-C (*n* = 290), no. (% of total)
Male	87 (61.3%)	79 (53.4%)	.19	1.38 (0.87, 2.0)	166 (57.2%)
Female	55 (38.7%)	69 (46.6%)	.19	0.7 (0.45, 1.15)	124 (42.8%)
Median age, y (IQR)	9.49 (7.07, 13.38)	8.62 (5.11, 11.59)	.006∗∗		9.26 (5.91, 12.58)
Race/ethnicity					
White	75 (52.8%)	105 (71.0%)	.002∗	0.46 (0.28, 0.74)	180 (62%)
Black or African American	50 (35.2%)	30 (20.3%)	.006∗	2.82 (1.26, 3.63)	80 (27.6%)
Asian	10 (7.0%)	8 (5.4%)	.63	1.38 (0.51, 3.46)	18 (6.2%)
Hispanic or Latino	57 (40.1%)	76 (51.4%)	.06	0.64 (0.4, 1.01)	133 (45.9%)
Other	10 (7.0%)	10 (6.6%)	.72	1.05 (0.42, 2.59)	20 (6.9%)
Comorbidities					
Asthma	13 (5.4%	2 (1.4%)	.003∗	7.36 (1.63, 33.2)	15 (5.2%)
Obesity	12 (8.5%)	9 (6.1%)	.50	1.42 (0.58, 3.5)	21 (7.2%)
Diabetes	1 (0.7%)	1 (0.7%)	.99	1.04 (0.06, 16.83)	2 (0.6%)
Previously healthy	118 (83.1%)	129 (87.2%)	.41	0.72 (0.38, 1.39)	247 (85.2%)

Note. Percentages may not sum up to 100 due to rounding. Race and ethnic group were self-reported. Subcategories in race and comorbidity categories are not mutually exclusive. Continuous variables are presented as median, interquartile range (IQR). Categorical variables are presented as percent frequency (%).∗*P* < .05, ∗∗*P* < .01.

*CI*, Confidence interval; *IQR*, interquartile range; *MIS-C*, multisystem inflammatory syndrome in children; *No*, number.

The median length of stay in the hospital was 6.56 days (IQR, 4.9, 8.2 days), and 73.8% (214/290) of the children required a median of 3.2 days (IQR, 2.0, 4.7 days) of intensive care unit (ICU) care (Table II). The majority of patients exhibited gastrointestinal (83.1%, 241/290), mucocutaneous (81.7%, 237/290), hematological (69%, 200/290), and cardiac (77.6%, 225/290) symptoms. The most frequently observed mucocutaneous manifestations were conjunctivitis (74.7%, 177/237), rash (62.4%, 148/237), dry or inflamed lips (39.7%, 94/237), and edema and erythema in the hands and feet (17.3%, 41/237). Regarding treatment, 95.9% (278/290) of patients received intravenous steroids, while aspirin (73.8%, 214/290), intravenous immunoglobulin (66.2%, 192/290), and anakinra (64.1%, 186/290) were also commonly administered. In addition, 20.7% (60/290) of children required continuous positive airway pressure/bi-level positive airway pressure, and 13.1% (38/290) of the more severely affected children needed mechanical ventilation. Only a small percentage of children (1.7%, 5/290) required extracorporeal membrane oxygenation.

**Table II:** Clinical course of MIS-C patients with and without a rash

Disease course	MIS-C without rash involvement (*n* = 142), no. (%)	MIS-C with rash involvement (*n* = 148), no. (%)	*P* value	Odds ratio (95% CI)	All MIS-C (*n* = 290), (% of total)
Length of stay, d (IQR)	6.71 (4.88, 8.37)	6.45 (5.02, 8.20)	.75		6.56 (4.9, 8.2)
Length of ICU stay, d (IQR)	3.24 (1.97, 4.73)	3.14 (1.92, 4.60)	.57		3.20 (2.0, 4.7)
Clinical requirements					
ICU care	104 (73.2%)	110 (74.3%)	.99	0.95 (0.56, 1.6)	214 (73.8%)
ECMO	2 (1.4%)	3 (2.0%)	.99	0.69 (0.11, 4.19)	5 (1.7%)
Ventilator	23 (16.2%)	15 (10.1%)	.16	1.71 (0.85, 3.44)	38 (13.1%)
CPAP/BIPAP	32 (22.5%)	28 (18.9%)	.47	1.25 (0.7, 2.2)	60 (20.7%)
Inotropes	73 (51.4%)	61 (41.2%)	.1	1.51 (0.95, 2.4)	134 (46.2%)
Shock	97 (68.3%)	85 (57.4%)	.07	1.6 (0.99, 2.58)	182 (62.8%)
Organ system involvement					
Cardiac	118 (83.1%)	107 (72.3%)	.03∗	1.88 (1.07, 3.32)	225 (77.6%)
Renal	47 (33.1%)	37 (25.0%)	.16	1.48 (0.89, 2.47)	84 (29.0%)
Respiratory	49 (34.5%)	44 (29.7%)	.45	1.25 (0.76, 2.04)	93 (32.1%)
Hematologic	104 (73.2%)	96 (64.9%)	.13	1.48 (0.9, 2.45)	200 (69.0%)
Gastrointestinal	117 (82.4%)	124 (83.8%)	.76	0.91 (0.49, 1.67)	241 (83.1%)
Neurologic	37 (26.1%)	41 (27.7%)	.9	0.92 (0.55, 1.55)	78 (26.9%)
Lab values, median (IQR)					
CRP (mg/dL)	20.4 (12.25, 26.6)	19.2 (13.52, 24.45)	.47		19.70 (13.3, 25.63)
ESR (mm/h)	50.5 (36.5, 64)	46 (30.25, 76)	.77		50 (33.5, 71.5)
Fibrinogen (mg/dL)	578.5 (472.25, 669)	573 (493, 669)	.87		575 (479, 669)
Procalcitonin (ng/mL)	4.21 (1.64, 18.15)	4.03 (1.7, 10.88)	.25		4.06 (1.7,15.37)
D-dimer (ug/mL)	3.73 (2.86, 5.78)	3.6 (2.46, 5.42)	.1		3.63 (2.7, 5.67)
Ferritin (ng/mL)	403.5 (259, 850)	370 (248, 823.5)	.55		382.5 (239, 727)
LDH (IU/L)	328 (269, 465)	328.5 (269, 446)	.95		328 (269, 454)
Troponin (ng/mL)	0.11 (0.04, 0.37)	0.07 (0.02, 0.20)	.02∗		0.09 (0.03, 0.28)
BNP (pg/mL)	512.1 (235.8, 1011.8)	529.9 (223.5, 853.6)	.55		532.75 (231.3, 951.8)
Absolute neutrophil Count (10^3^/uL)	11.45 (8.22, 16.58)	11.73 (7.62, 16.23)	.55		11.54 (8.01, 16.27)
Platelet count (10^3^/uL)	383.5 (282, 497)	381 (271.25, 496)	.94		382.5 (278.75, 494.75)
Absolute lymphocyte count (10^3^/uL)	0.76 (0.51, 1.19)	0.69 (0.43, 1.23)	.31		0.73 (0.45, 1.19)
Albumin (g/dL)	2.8 (2.5, 3.3)	2.8 (2.5, 3.3)	.92		2.8 (2.5, 3.3)
Treatment course					
IVIG	89 (62.7%)	103 (69.6%)	.22	0.73 (0.45, 1.2)	192 (66.2%)
IV steroids	135 (95.1%)	143 (96.6%)	.83	0.67 (0.21, 2.18)	278 (95.9%)
Anakinra	94 (66.2%)	92 (62.2%)	.54	1.19 (0.74, 1.93)	186 (64.1%)
Broad-spectrum antibiotics	73 (51.4%)	65 (43.9%)	.24	1.35 (0.85, 2.14)	138 (47.6%)
Aspirin	106 (74.7%)	108 (73.0%)	.79	1.09 (0.65, 1.84)	214 (73.8%)

Percentages may not sum up to 100 due to rounding. Continuous variables are presented as median and interquartile range (IQR). Categorical variables are presented as percent frequency (%). ∗*P* < .05.

*BNP*, B-type natriuretic peptide; *CI*, confidence interval; *CPAP/BIPAP*, continuous positive airway pressure/bilevel positive airway pressure; *CRP*, C-reactive protein; *ECMO*, extracorporeal membrane oxygenation; *ESR*, erythrocyte sedimentation rate; *ICU*, intensive care unit; *IQR*, interquartile range; *IV*, intravenous; *IVIG*, intravenous immunoglobulin; *LDH*, lactate dehydrogenase; *MIS-C*, multisystem inflammatory syndrome in children; *No*, number.

To determine whether the presence of a rash was associated with specific clinical features or an altered clinical course, we analyzed the data from MIS-C patients who developed a rash and found 51% (148/290) of patients developed a rash (Table II). Asthma was observed in a higher percentage of MIS-C patients without a rash (5.4% vs 1.4%, *P* = .003) and was the only significant comorbidity difference between groups.

We found a statistically significant age discrepancy (*P* = .006) with a median age of 8.62 (IQR, 5.11, 11.59) in MIS-C children with a rash, while the median age of children without a rash was 9.49 (IQR, 7.07, 13.38). Of the children who developed a rash, 71% (105/148) were White, while White children had lower odds of presenting with no rash (odds ratio (OR) [95% CI]: 0.46 [0.28, 0.74], *P* = .002). In contrast, the percentage of Black or African American children was lower in the rash-positive group (20.3%, 30/148) and had higher odds of presenting without a rash (OR [95% CI]: 2.82 [1.26, 3.63], *P* = .006) (Table I). These demographic differences provide insight into a potential association of race and ethnicity in the development of specific cutaneous manifestations in MIS-C children.

Regarding organ system involvement, we found that cardiac involvement was more prevalent in pediatric patients without a rash (83.1%, 118/142) compared to those with a rash (72.3%, 107/148) (OR [95% CI]: 1.88 [1.07, 3.32], *P* = .03). We also observed a statistically significant difference in the maximum troponin levels between the 2 patient groups, with those with a rash having a lower troponin level (0.07; IQR, 0.02-0.20) than those without a rash (0.11; IQR, 0.04-0.37) (*P* = .02).

Our analysis also revealed a positive relationship between both troponin levels and cardiac involvement in MIS-C patients with increasing age. The 0- to 5-year-old cohort had the lowest peak troponin level (0.04; IQR, 0.01-0.07) and the lowest percentage of cardiac involvement (73.2%), while the 12- to 21-year-old cohort had the highest peak troponin level (0.23; IQR, 0.08, 0.98) and the highest percentage of cardiac involvement (84.0%). When patients were further stratified by age cohorts, differences in troponin levels between the rash and nonrash cohorts persisted but did not reach statistical significance likely due to diminished power from lower sample size (data not shown).

**Fig 1:**
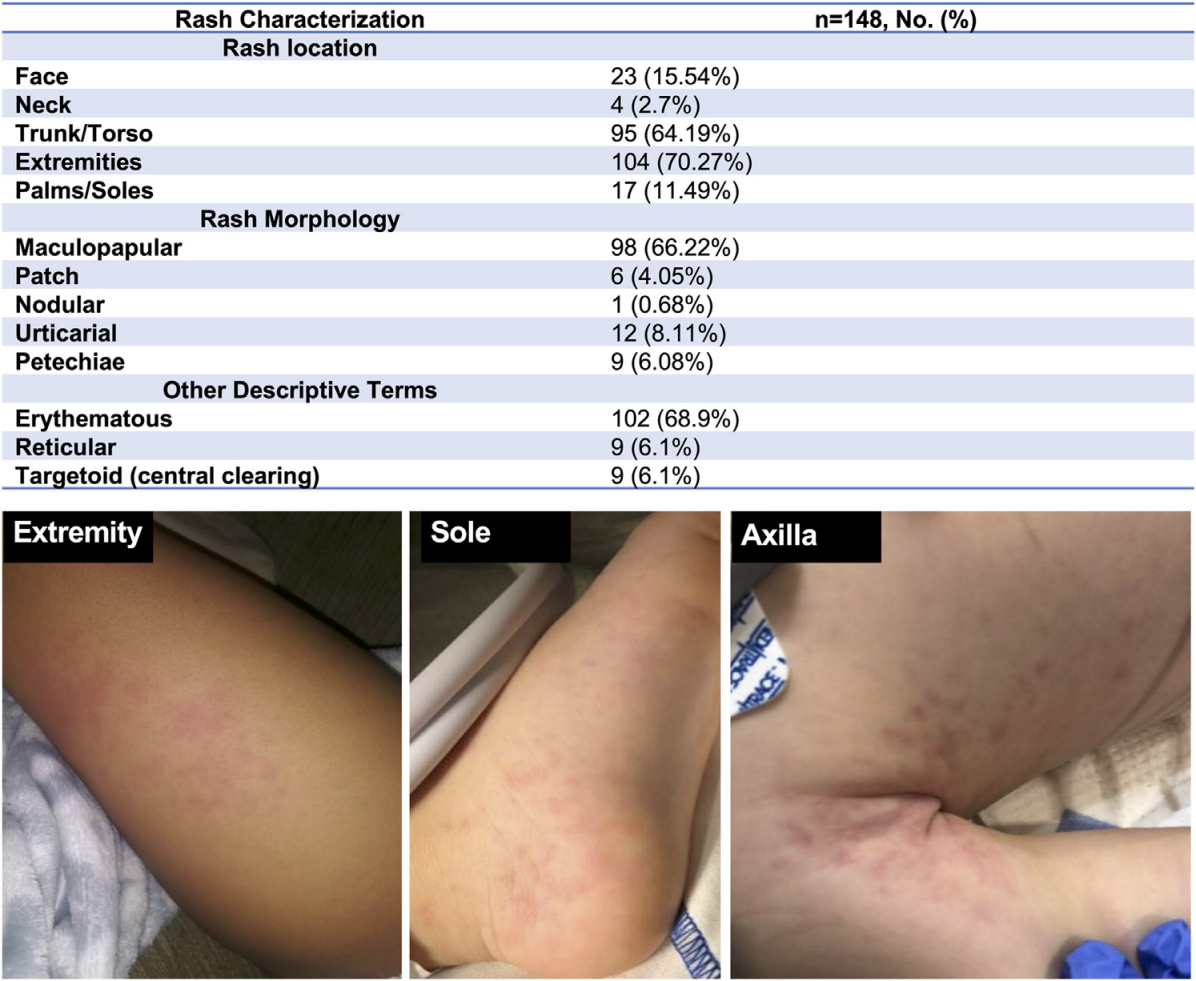
Rash characterization with patient images demonstrating the common location and morphology of the rash observed in MIS-C patients. The percentage of patients presenting with a rash in the given location and morphology is shown. Percentages may not sum up to 100 due to rounding. Categorical variables are presented as percent frequency (%). Patient images of the rash observed in the extremity, sole, and trunk are provided as representative images. *MIS-C*, Multisystem inflammatory syndrome in children; *No*, number.

Fig 1 summarizes the types of rashes that were observed in children with MIS-C. We found that the most common type of rash was an erythematous (68.9%, 102/148) and maculopapular (66.2%, 98/148) rash, accounting for the majority of rashes observed, with other presentations being much less frequent. The majority of rashes were localized to either the extremities (70.3%, 104/148) or the torso region (64.2%, 95/148). Clinical images from our patient cohort of the most commonly observed rash on the extremity, sole, and trunk are shown in Fig 1.

## Discussion

MIS-C is a rare but severe condition that has emerged during the COVID-19 pandemic. We analyzed the demographic background, clinical course, inflammatory markers, and treatment approach in a large cohort of MIS-C patients, a significant portion of whom required care in the ICU. Fifty-one percent of patients admitted to the ICU were found to have a rash, which corresponds to the overall percentage of patients observed with a rash (51%), indicating that rash manifestations were consistently diagnosed regardless of the clinical severity. We aimed to add to the accumulating knowledge on MIS-C disease presentation and, in particular, parse out the clinical sequela of MIS-C patients with rash manifestations.

Prior studies on the association between mucocutaneous symptoms and disease severity in MIS-C suggest that mucocutaneous manifestations are associated with a less severe disease course.^8^^,^^9^ Rao et al reported troponin levels were negatively associated with mucocutaneous symptoms.^9^ In our study, we stratified patients based on the presence of cutaneous findings in the form of a rash and found a negative correlation between the presence of a rash and troponin level peaks.

Among the patients included in our study, 51% of those diagnosed with MIS-C displayed a rash as part of their mucocutaneous involvement, while 82% presented with any mucocutaneous symptom (ie, cutaneous, tongue, lip, eye involvement). We subsequently categorized all patients based on the presence or absence of a rash. Our analysis revealed that younger children were more likely to develop a rash in conjunction with MIS-C. Additionally, we observed that white children had a higher frequency of rashes compared to Black/African-American children. The association between race and the presence of a rash in MIS-C remains unclear.

MIS-C children without a rash had a statistically significant increase in maximum troponin levels (0.11 vs 0.07) and cardiac involvement (83% vs 72.3%). The 99th percentile for the normal reference range of our troponin test is <0.03 ng/mL, and troponin levels higher than 0.03 ng/mL are concerning for MIS-C-related myocardial injury, requiring close monitoring by cardiologists and treatment with aspirin, intravenous immunoglobulin, and inotropes. The mechanism by which cutaneous manifestations in the form of a rash may be negatively associated with cardiac involvement and increased troponin levels in MIS-C is not known, but the immunoinflammatory mechanism that drives this association is an area of interest for future research. Aside from the higher troponin levels and increased cardiac involvement, the length of hospital stay or clinical course was not statistically different in MIS-C patients who developed a rash.

Finally, we analyzed the characteristics and location of the rash observed in our studied cohort. The majority of patients with a rash exhibited a blanching, erythematous, and/or maculopapular rash on the trunk and/or extremities. Urticaria and maculopapular rashes were the most commonly observed rash in previous reports.^7^^,^^10^

Overall, this descriptive retrospective cohort study of MIS-C patients diagnosed at TCH provides a robust analysis of some of the data points previously connected to MIS-C with this study having the advantage of being one of the largest studies to date. However, there are some limitations to this study. A notable limitation is the absence of input from a dermatologist in characterizing the cutaneous manifestations of every patient. Though many of the MIS-C patients were followed by pediatric dermatologists, there is a risk of rash mischaracterization or incomplete documentation in patients who presented with severe organ complications that overshadowed the cutaneous manifestations. With this being a large retrospective analysis the temporal associations between the rashes and inflammatory markers, organ system, and clinical outcomes were unable to be established. The statistically significant difference in self-identified African American/Black and White patients presenting with rash manifestations raises a potential limitation of the underdiagnosis of rash manifestations in skin of color patients. Another limitation is that this data may not offer a comprehensive clinical overview of MIS-C since the majority of cases included in the study required ICU treatment (73.8%). Consequently, the presentation of the condition might be biased toward more severe symptoms. This can limit our understanding of the full spectrum of MIS-C disease clinical findings and outcomes.

## Conflicts of interest

None disclosed.
